# High‐Performance Li‐O_2_ Batteries with Trilayered Pd/MnO*_x_*/Pd Nanomembranes

**DOI:** 10.1002/advs.201500113

**Published:** 2015-05-26

**Authors:** Xueyi Lu, Junwen Deng, Wenping Si, Xiaolei Sun, Xianghong Liu, Bo Liu, Lifeng Liu, Steffen Oswald, Stefan Baunack, Hans Joachim Grafe, Chenglin Yan, Oliver G. Schmidt

**Affiliations:** ^1^ Institute for Integrative NanosciencesLeibniz Institute for Solid State and Materials Research DresdenHelmholtz Strasse 20Dresden01069Germany; ^2^ Materials Systems for NanoelectronicsChemnitz University of TechnologyReichenhainer Strasse 70Chemnitz09107Germany; ^3^ International Iberian Nanotechnology LaboratoryBraga4715‐330Portugal; ^4^ Institute for Complex MaterialsLeibniz Institute for Solid State and Materials Research DresdenHelmholtz Strasse 20Dresden01069Germany; ^5^ Institute for Solid State ResearchLeibniz Institute for Solid State and Materials Research DresdenHelmholtz Strasse 20Dresden01069Germany; ^6^ College of PhysicsOptoelectronics and Energy and Collaborative Innovation Center of Suzhou Nano Science and TechnologySoochow UniversitySuzhou215006China; ^7^ Center for Advancing Electronics DresdenDresden University of TechnologyDresden01069Germany; ^8^ Merge Technologies for Multifunctional Lightweight StructuresChemnitz University of TechnologyChemnitz09107Germany

**Keywords:** cathodes, Li‐O_2_ batteries, catalysts, trilayered nanomembranes

## Abstract

**Trilayered Pd/MnO_*x*_/Pd nanomembranes** are fabricated as the cathode catalysts for Li‐O_2_ batteries. The combination of Pd and MnO_*x*_ facilitates the transport of electrons, lithium ions, and oxygen‐containing intermediates, thus effectively decomposing the discharge product Li_2_O_2_ and significantly lowering the charge overpotential and enhancing the power efficiency. This is promising for future environmentally friendly applications.

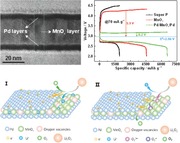

Environmental pollution and fossil fuel depletion have led to massively increased exploitation of new types of energy, including nuclear, solar, and wind power source.^1^, ^2^ Lithium ion batteries (LIBs), in turn, developed over the last two decades dominate the portable electronics market as the power sources.^3^ However, with the ever‐increasing power and energy needs especially for low‐emission electric vehicles (EVs), LIBs still cannot reach the specific level of transportation (a driving range of 500 km within one charge^1^ because of their low energy densities.^1^, ^4^) New concept lithium batteries have triggered worldwide interest these years, including aprotic and aqueous Li‐O_2_ batteries and Li‐Se batteries. Nonaqueous Li‐O_2_ batteries first reported in 1996^5^ possess exceptionally high energy density which is close to that of gasoline,^3^, ^6^ much higher than that of LIBs.^7^ The operation mechanism of nonaqueous Li‐O_2_ batteries is mainly based on the reaction 2Li + O_2_ ⇌ Li_2_O_2_,^8^ the forward of which represents the discharge while the reverse describes the charge process.^9^ On discharging, the oxygen is reduced to form Li_2_O_2_ 10 that has a poor conductivity and is insoluble in the nonaqueous electrolyte and therefore can easily block the pores of carbon materials,^11^ resulting in large polarization and high overpotential. It is such overpotential that has limited current Li‐O_2_ batteries to a few discharge–charge cycles and poor power efficiency that restrict the practical use of Li‐O_2_ batteries.

Extensive research efforts have been dedicated to addressing above issues, mainly focusing on the cathode catalysts (including carbon materials, precious metals, metal oxides, and nonprecious metals^12^ that can accelerate the formation and decomposition of Li_2_O_2_ and lower the overpotential of both the oxygen reduction reaction (ORR) and oxygen evolution reaction (OER),^13^ thus improving the performance of batteries in the meanwhile. Owing to their special advantages such as abundance, environmental friendliness, and low cost,^14^ transition metal oxides have been broadly applied in electric devices that include supercapacitors,^15^, ^16^ LIBs,^17^ and Li‐O_2_ batteries.^18^ However, the low conductivity of such oxides has limited their performance to some extent.^19^

In this work, we report the fabrication of trilayered Pd/MnO*_x_*/Pd nanomembranes prepared by rolled‐up technology (Figure S1, Supporting Information).^20^ Rolled‐up technology has recently proven to be an efficient method on micro/nanoscale for promising future applications. With rolled‐up technology, it is convenient to prepare single‐layered or multilayered composite membranes with well‐controlled thicknesses, stacking sequence, and chemical composition.^21^ Moreover, structure stability can be maintained by self‐winding to release the intrinsic strain accommodated in the membranes.^21^ With such new type of hybrid cathode configuration based on trilayered Pd/MnO*_x_*/Pd nanomembranes, the power efficiency of Li‐O_2_ batteries was greatly improved from ≈60% to ≈86%, as compared to the bare MnO*_x_* nanomembranes. Notably, by sandwiching the MnO*_x_* layer in between two ultrathin Pd films, the charge overpotential was significantly lowered to ≈0.2 V with such a hybridized Pd/MnO*_x_*/Pd nanomembranes cathode, thus reaching the theoretical limit proposed in the literature.^22^

The morphology of Pd/MnO*_x_*/Pd nanomembranes was characterized by scanning electron microscopy (SEM). A curved nanostructured Pd/MnO*_x_*/Pd membrane with a diameter of 10 μm and nanometer‐scale thickness is displayed in **Figure** **1**a, which features a hollow body and layered windings. With the purpose of confirming the sandwiched structure and measuring the exact thicknesses of the layers, we deposited a Pd/MnO*_x_*/Pd stack on a Si substrate without photoresist. For TEM investigation a lamella was prepared by focused ion beam (FIB) cutting (Zeiss NVision40). Figure 1b shows the polycrystalline MnO*_x_* layer (≈23 nm wide) between two smooth Pd layers (4–5 nm wide) on the Si substrate (with ≈3 nm native silicon oxide). Upon a close look at the nanomembrane (Figure 1c), we can see that the membrane consists of a great number of small grains that contribute to the polycrystalline structure of the thin film, which is in agreement with the result of Figure 1b. The high‐resolution transmission electron microscopy (HR‐TEM) image (Figure 1d) shows that the thin Pd/MnO*_x_*/Pd nanomembranes are composed of Pd and MnO*_x_*. The lattice spacing of 0.19 nm clearly revealed in Figure 1d may correspond to Pd (200) or MnO_2_ (210). And the lattice spacing of 0.22 nm might correspond to Pd (111) or MnO_2_ (200) or MnO (111) because they have similar lattice distances. Similarly, the lattice spacing of 0.31 nm may correspond to MnO_2_ (110) or Mn_3_O_4_ (−103).^23^ These results indicate that manganese oxides besides MnO_2_ used for the deposition source might exist in the nanomembranes, which can be attributed to the oxygen vacancies during the materials deposition.

**Figure 1:**
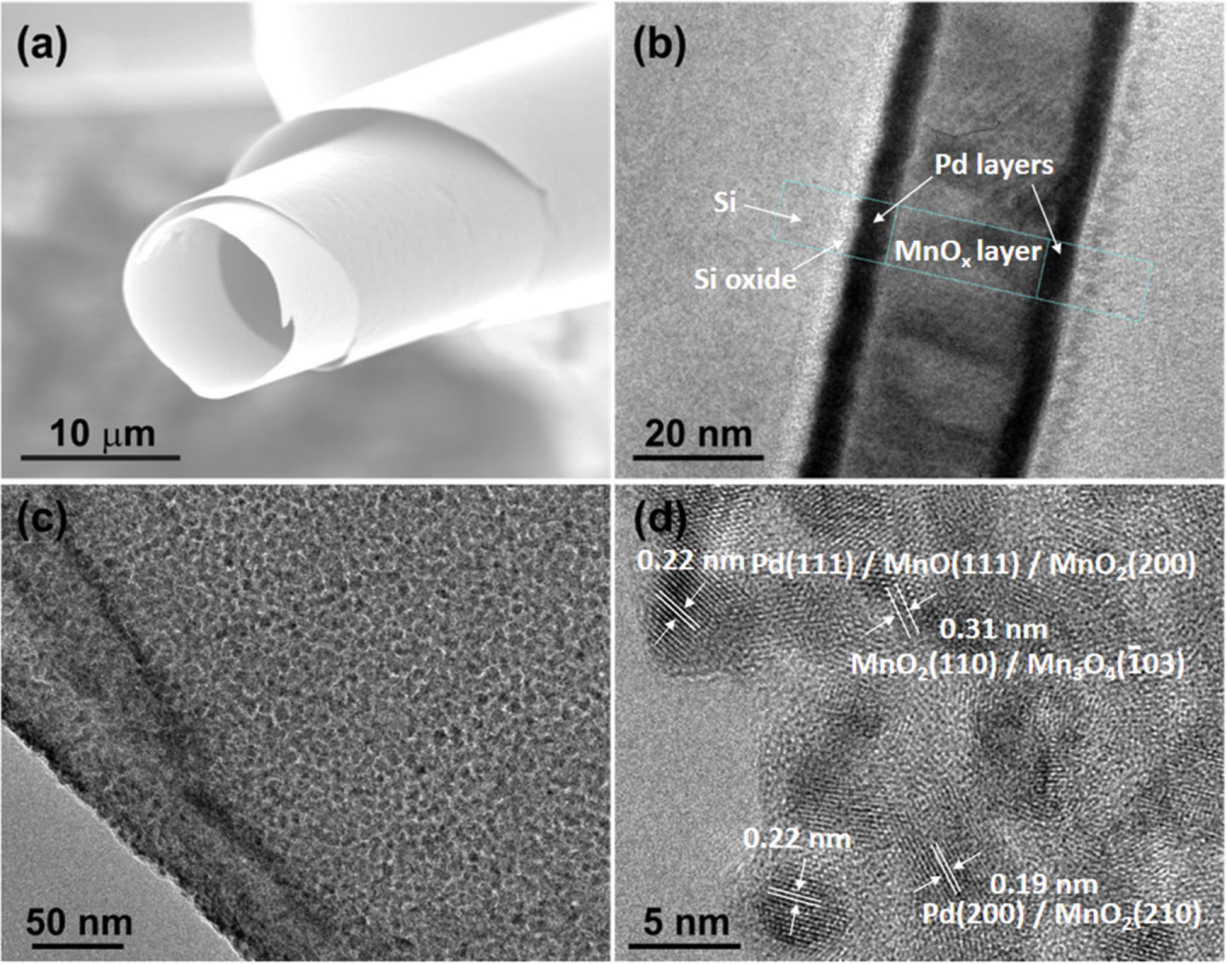
a) SEM image of a typical Pd/MnO*_x_*/Pd nanomembrane. b) TEM image of trilayered Pd/MnO*_x_*/Pd nanomembrane on a Si substrate. c,d) HR‐TEM images of the Pd/MnO*_x_*/Pd nanomembrane.

In order to determine the chemical composition, the as‐prepared Pd/MnO*_x_*/Pd nanomembranes were characterized by X‐ray photoelectron spectroscopy (XPS). The wide‐scan XPS spectrum of the material is shown in **Figure** **2**a. Figure 2b,c show the detailed XPS spectra of Pd 3d and Mn 2p of the Pd/MnO*_x_*/Pd nanomembranes. Each spectrum was analyzed using peak‐fit procedures. The peaks located at 334.9 and 335.8 eV correspond to Pd and PdO and the content of PdO is high which comes from two aspects. The high PdO content originates from either the naturally oxidized surface layer of the Pd nanomembranes or the oxidation products arising from the reaction between Pd and MnO*_x_*. From the appearance of high energy satellite peaks (around 647 and 658.5 eV) in Figure 2c, which are typical for MnO,^24^ we conclude that MnO is the major phase existing in the nanomembranes or at least near the MnO*_x_*/Pd interface. This may result from incomplete oxidation of the MnO*_x_* during deposition or the reaction of MnO*_x_* with Pd during the sample preparation process. Raman spectroscopy was also used to investigate the composition of the as‐prepared samples (Figure 2d). The main peaks at ≈644 and ≈346 cm^−1^ correspond to the features of MnO*_x_* materials, including MnO_2_,^25^, ^26^ Mn_3_O_4_,^25^ and Mn_2_O_3_,^27^ indicating there may exist different types of manganese oxides in the hybrid nanomembranes, which is in agreement with the result of HR‐TEM and our previous report.^16^ Due to the generation of manganese oxides with lower oxygen percentages in comparison with MnO_2_ used for the deposition source (including MnO and possible Mn_2_O_3_ and Mn_3_O_4_), which is caused by oxygen vacancies or interaction between Pd and MnO*_x_*, we labeled our prepared material as Pd/MnO*_x_*/Pd (*x* < 2). The weight percentages of Pd and MnO*_x_* in the Pd/MnO*_x_*/Pd nanomembranes are displayed in Figure S2 (Supporting Information).

**Figure 2:**
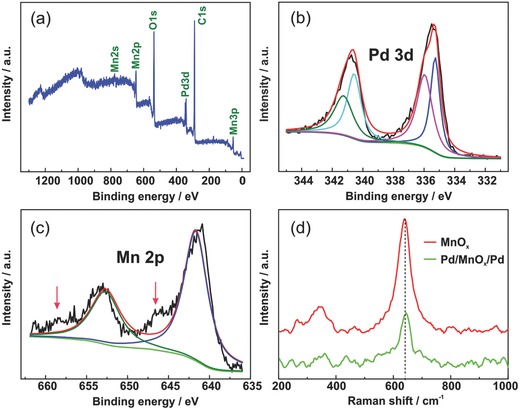
a–c) XPS spectra of Pd/MnO*_x_*/Pd nanomembranes. d) Raman spectra of MnO*_x_* and Pd/MnO*_x_*/Pd nanomembranes.

Cyclic voltammetry was performed on an assembled battery with Pd/MnO*_x_*/Pd cathode material from 2.0 to 4.5 V at a scan rate of 0.1 mV s^−1^ (Figure S3, Supporting Information). The curves show that the cathodic peaks during the first and second cycles are similar, while the anodic peaks are much different. The anodic peak during the first cycle appears at ≈4.0 V and it shifts negatively to ≈3.4 V during the second cycle, which is in agreement with a previous report.^3^ The behavior of the first cycle may be ascribed to an electro‐activation process of the freshly prepared electrodes, including the activation of catalyst, the diffusion of oxygen into the pores of carbon, as well as the immersion of the electrolyte into the whole electrode. We also performed a galvanostatic charging test to determine the stable voltage window of the LiCF_3_SO_3_/TEGDME used in our work. A Li‐O_2_ battery with Pd/MnO*_x_*/Pd electrode was assembled and charged directly from the open circuit without discharging where only electrolyte degradation occurs during the process. Figure S4 (Supporting Information) shows that the voltage keeps near constant at the beginning and increases sharply at 4.75 V, indicating that the electrolyte was decomposed severely when the voltage reached 4.75 V. Therefore, we chose 4.5 V as the charging set‐off in the following galvanostatic tests to avoid the decomposition of the electrolyte.

The Pd/MnO*_x_*/Pd and MnO*_x_* nanomembranes were used as the cathode catalysts for rechargeable Li‐O_2_ cells and the results are shown in **Figure** **3**. At a current density of 70 mA g^−1^, the Li‐O_2_ battery with Super P (carbon black) delivers a specific capacity of 1930 mAh g^−1^ with discharge and charge overpotential of 0.29 and 1.44 V (Figure 3a). The contribution of carbon paper can be neglected (Figure S5, Supporting Information). MnO*_x_* nanomembranes exhibit an increased capacity of 4500 mAh g^−1^ and lower overpotentials as compared to Super P. Notably, when Pd/MnO*_x_*/Pd nanomembranes were used as the cathode catalyst, the specific capacity is greatly enhanced up to 6570 mAh g^−1^ which is about 1.5 times and 3.4 times more than that of MnO*_x_* and Super P, respectively. More importantly, the charge potential is sharply lowered to only ≈3.16 V (the overpotential is ≈0.2 V, which is the theoretical limit proposed in the literature^22^), leading to a power efficiency of ≈86%. The Li‐O_2_ cell with Pd/MnO*_x_*/Pd still obtains 4200 mAh g^−1^ when the current density increases to 200 mA g^−1^ (Figure 3b), which is 1.6 times and 5.7 times that of MnO*_x_* and Super P, respectively. Even at a high current density of 200 mA g^−1^, the power efficiency of Pd/MnO*_x_*/Pd still stays over 80% (Figure 3c), much higher than those of bare MnO*_x_* and Super P. We also tested the Li‐O_2_ cells with Pd/MnO*_x_*/Pd electrodes at higher current densities and the result is shown in Figure 3d. At a current density of 1 A g^−1^, the charge potential is still lower than 3.8 V with a high power efficiency of about 65%. The presence of Pd nanomembranes plays an important role in improving the power efficiency and lowering the charge overpotential due to the electrocatalytic effect and the enhanced electronic transport properties. This claim is backed by a lower power efficiency and much higher charge overpotential obtained for the bare MnO*_x_* cathode without Pd.

**Figure 3:**
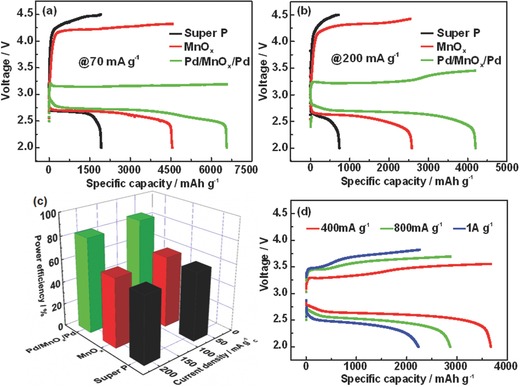
a,b) First discharge–charge curves of Li‐O_2_ batteries with Pd/MnO*_x_*/Pd or MnO*_x_* or super P electrodes at a current density of 70 and 200 mA g^−1^, respectively. c) Power efficiencies of Li‐O_2_ batteries with Pd/MnO*_x_*/Pd, MnO*_x_*, super P electrodes. d) Discharge−charge curves of Li‐O_2_ batteries with Pd/MnO*_x_*/Pd at different current densities.

In order to test the stability of Pd/MnO*_x_*/Pd electrode, the batteries were discharged and charged to a capacity of 2000 mAh g^−1^ at current densities of 70 (**Figure** **4**a,b) and 200 mA g^−1^ (Figure S6, Supporting Information) for five cycles. The voltage profiles in both Figure 4a,b show that the discharge and charge potential is ≈2.69 and ≈3.18 V, respectively, and the power efficiency remains constant at ≈84% during the whole charge–discharge process. At a current density of 200 mA g^−1^, the average discharge potential decreases to ≈2.55 V (Figure S6, Supporting Information), while the power efficiency still stabilizes at ≈80%. To further verify the stability of the Pd/MnO*_x_*/Pd nanomembranes, we cycled the batteries with both MnO*_x_* electrode and Pd/MnO*_x_*/Pd electrode at 500 mA g^−1^ and a limited capacity of 1000 mAh g^−1^ (Figure S7, Supporting Information, and Figure 4c,d). As can be seen from the voltage curves (Figure S7, Supporting Information), the battery with MnO*_x_* electrode only cycles 48 times before the discharge voltage drops to 2.0 V with the charge voltage keeping as high as 4.5 V. Surprisingly, there is no apparent degradation of both the discharge and charge potentials up to 190 cycles for the battery with Pd/MnO*_x_*/Pd electrode and it finally cycles 269 times before the discharge voltage drops to 2.0 V, which shows a great improvement than usually reported in the literature. Moreover, the terminal charge potential of Pd/MnO*_x_*/Pd electrode keeps below 4.2 V in the whole process, effectively suppressing the side reactions and achieving stable cycling performance. The discharge voltage declines rapidly from about 250 cycles preceded by a slow voltage increase during the charge cycle, which may be caused by the consumption of Li foil, depletion of the electrolyte, and also possible degradation of the membranes electrode after the long‐time cycling.

**Figure 4:**
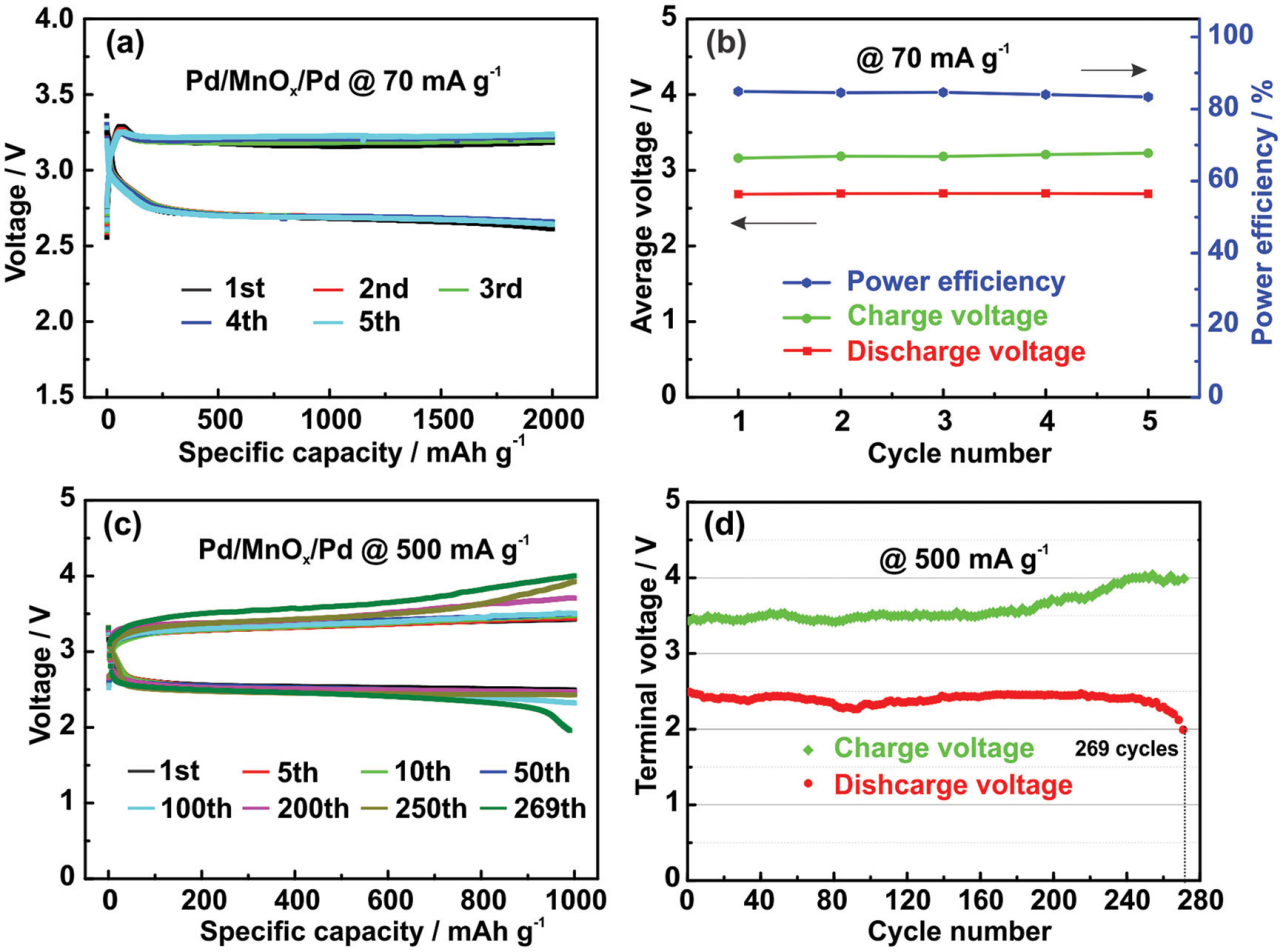
a,b) Discharge−charge curves and power efficiency of Li‐O_2_ battery with Pd/MnO*_x_*/Pd cathode at 70 mA g^−1^ under a specific capacity limit of 2000 mAh g^−1^. c,d) Discharge−charge curves and terminal voltages of Li‐O_2_ battery with Pd/MnO*_x_*/Pd at 500 mA g^−1^ under a specific capacity limit of 1000 mAh g^−1^.

We have examined the discharge products of both MnO*_x_* and Pd/MnO*_x_*/Pd electrodes by SEM and XRD. As shown in Figure S8a,b (Supporting Information), near amorphous Li_2_O_2_ was formed on the MnO*_x_* electrode, which is different from the toroid‐like shaped discharge product usually reported in the literature.^28^ Unlike MnO*_x_* electrode, canoes shaped Li_2_O_2_ particles with length of ≈3 μm and width of ≈1.6 μm were formed on Pd/MnO*_x_*/Pd electrode after the first discharge process (**Figure** **5**a), which are also different from the toroid‐like morphology. The incorporated Pd nanomembranes offer numerous surface sites for the nucleation of crystalline Li_2_O_2_. The Pd/MnO*_x_*/Pd electrodes after first charge, tenth discharge, and tenth charge process were also characterized by SEM (Figure 5b–d). As can be seen from Figure 5b, the discharge products Li_2_O_2_ completely disappear after the first charge. Similarly, canoes shaped Li_2_O_2_ exhibit again after the tenth discharge which is completely decomposed after the following charge procedure, indicating the Pd/MnO*_x_*/Pd electrode possesses efficient reversibility. We also tested the pristine Pd/MnO*_x_*/Pd electrode and the electrode after the first discharge and first charge by XRD. As shown in Figure S9 (Supporting Information), the data of electrode after first discharge match well to the reference XRD pattern of Li_2_O_2_ (powder diffraction file number 01‐073‐1640). No other major discharge products except Li_2_O_2_ are observed. The spectrum of the electrode after charging is nearly the same as the pristine electrode, indicating that the discharge product Li_2_O_2_ has been completely decomposed and there are rare byproducts caused by electrolyte degradation during charging or at least in the first charge process. To further check the change of the products on the electrode, the electrode after discharge and charge was characterized by ^7^Li NMR (nuclear magnetic resonance) (Figure S10, Supporting Information). The signal of discharge electrode is in good agreement with pure Li_2_O_2_ reported in the literature,^29^ indicating that Li_2_O_2_ is the major discharge product. After charging, the Li_2_O_2_ was completely decomposed, leaving one Li‐containing product with much lower intensity and lower frequency (maximum at ≈ −40 ppm) which may not be detected by XRD because of its low content and the equipment limit.

**Figure 5:**
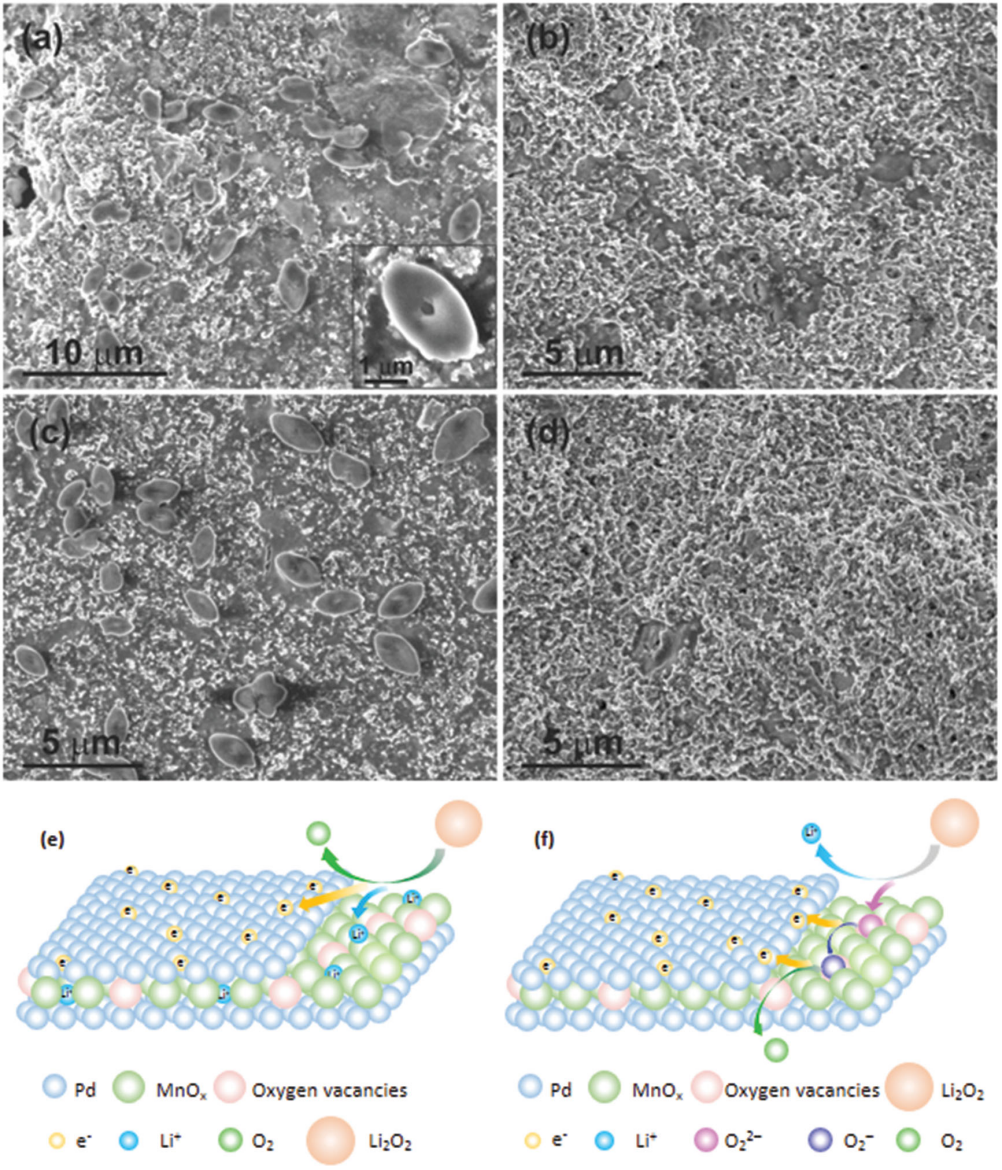
SEM images of Pd/MnO*_x_*/Pd electrode after a) first discharge, b) first charge, c) tenth discharge, and d) tenth charge. e,f) Proposed possible synergistic effect between Pd and MnO*_x_* in Pd/MnO*_x_*/Pd nanomembranes.

We suppose that the extremely low charge overpotential, high stability, and good reversibility can be ascribed to the particular sandwiched structure of Pd/MnO*_x_*/Pd nanomembranes and the synergistic catalytic effect between Pd and MnO*_x_* in the cathode. To begin with, the trilayered Pd/MnO*_x_*/Pd nanomembranes fabricated by rolled‐up technology during film strain release could effectively reduce the system energy, thereby enhancing the tolerance to stress cracking during cycling. Moreover, the nanomembranes are composed of grains instead of nanoparticles; therefore, the common aggregation phenomenon of nanoparticles does not exist in the nanomembrane materials, avoiding the sharp decrease of the catalytic active sites caused by aggregation. In addition, the synergistic effect between Pd and MnO*_x_* plays a momentous role in the charge process.

We propose two possible mechanisms for the synergy (Figure 5e,f). The noble metal Pd has a good electronic conductivity and the Li^+^ can easily insert into MnO*_x_* 30 that is commonly used for lithium storage. Therefore, the Pd/MnO*_x_*/Pd nanomembranes provide two “highways” in the charge procedure—one for fast electron transport and the other for fast Li^+^ diffusion (Figure 5e). Due to their synergistic effect, Li_2_O_2_ can be decomposed more easily than single‐component MnO*_x_*. Furthermore, Pd/MnO*_x_*/Pd nanomembranes prepared by electron beam evaporation possess numerous oxygen vacancies confirmed by the formation of manganese oxides with lower oxygen percentages in comparison with MnO_2_ used for the deposition source (including MnO and possible Mn_2_O_3_ and Mn_3_O_4_), which has been confirmed by the HR‐TEM, XPS, and Raman test results. These oxygen vacancies can facilitate binding oxygen‐containing intermediate O_2_^2−^ to the catalyst accompanied by the cleavage of Li^+^ from Li_2_O_2_.^31^ Pd accelerates the electrons transport in the following steps—the conversion from O_2_^2−^ to O_2_^−^ and the formation of O_2_ from the oxidation of O_2_^−^ which is also easily absorbed by oxygen vacancies (Figure 5f). It is the synergistic effect that facilitates the OER, effectively promoting the performances of Li‐O_2_ batteries especially significantly lowering the charge potential from ≈4.26 to ≈3.16 V. Additionally, the nanomembrane consisting of two layers of highly catalytic Pd films and one layer of MnO*_x_* film to form a novel trilayered structure provides stable conductive networks and large contacting areas between Pd and MnO*_x_*, greatly enhancing the synergistic catalytic effect.

In summary, we have fabricated novel trilayered Pd/MnO*_x_*/Pd nanomembranes through a strain release method. The unique trilayered Pd/MnO*_x_*/Pd nanomembranes as cathodes for Li‐O_2_ batteries offer significantly lowered charge overpotential of ≈0.2 V, greatly enhanced power efficiency of ≈86%, and a stable cycle life up to 269 cycles. We expect this new architecture can pave the way for the future development of Li‐O_2_ cathode materials with high performance for the application of low‐emission electric vehicles.

## Experimental Section

*Materials Preparation*: The fabrication process of trilayered Pd/MnO*_x_*/Pd nanomembranes is depicted in Figure S1 (Supporting Information). In a typical procedure, a very thin photoresist (AR P3510) as the sacrificial layer was first spin‐coated onto an aluminum foil (thickness of 13 mm) coated silicon wafer at a speed of 3500 rpm for 15 s. Then, the aluminum coated silicon wafer was baked at 90 °C on a hotplate for 5 min to dry the photoresist. After that, 3 nm Pd, 30 nm MnO*_x_*, and 3 nm Pd were sequentially deposited onto the photoresist by electron beam evaporation. After MnO*_x_* deposition, the chamber was evacuated for 20 min to reduce the oxygen partial pressure and to avoid the oxidation of Pd. Once the deposition was finished, the aluminum foil was unwrapped from the Si wafer and immersed into acetone to remove the photoresist. Owing to the strain release, the membranes peeled off from the substrate and rolled up automatically. Finally, the sample was filtered and dried in a critical point dryer (CPD) to avoid the collapse of the tubes. For comparison, bare MnO*_x_* nanomembranes were also prepared using the same method.

*Materials Characterization*: The composition of the prepared materials was checked by Raman spectroscopy (from Renishaw) with laser wavelength of 442 nm and XPS spectrometer (PHI 5600) using monochromatized Al Kα X‐rays (300 W) and a pass energy of 29 eV. For SEM images, Zeiss DSM982 (Gemini, Germany) was operated at 5 kV to characterize the samples. The microstructure and composition were also investigated by TEM (FEI Titan ChemiSTEM 80‐200) at 200 keV and CM20 FEG (Philips) at 200 keV. The electrode disc before and after charging was tested by X‐ray diffraction (XRD, PANalyticalX'Pert PRO Diffraction, Co Kα radiation). ^7^Li NMR test was measured in a field of 9.0934 T at room temperature (293 K), corresponding to zero shift at a frequency of 150.46 MHz (the gyromagnetic ratio of 7Li: *γ* = 16.5461 MHz T^−1^).

*Battery Measurements*: The electrochemical test was conducted by a Swagelok‐type battery that comprises a lithium foil anode and a nanomembrane‐contained cathode, Whatman glass fiber as the separator, 1 m LiCF_3_SO_3_ in TEGDME as the electrolyte. The cathode was composed of carbon black (Super P), catalyst materials, and polyvinylidene fluoride (PVDF) with the mass ratio of 60:20:20 in 1‐methyl‐2‐pyrrolidone (NMP). The carbon black was first mixed with PVDF/NMP solution to obtain homogenous slurry through ultrasonication. Then, the nanomembranes were added to the above slurry and mixed with it through vibration instead of ultrasonication to avoid the collapse of the membranes. After that, the mixture was spread onto the porous carbon paper and dried in a vacuum oven at 120 °C for 12 h. The mass of each electrode was measured to be 0.3–0.5 mg cm^−2^ after drying. The lithium oxygen batteries were assembled in an argon‐filled glove box (H_2_O < 0.1 ppm, O_2_ < 0.1 ppm, MBraun, Germany). During the test the batteries were sealed in 1 atm O_2_ in order to avoid the side influence of humidity and CO_2_. The galvanostatic discharge−charge tests were performed by an Arbin BT2000 system at various current densities. The current densities and capacities were normalized by the weight of the carbon. Cyclic voltammetry tests were carried out on a Zahner‐elektrik IM6 instrument (Germany) in the range of 2.0−4.5 V at room temperature.

## Supporting information

As a service to our authors and readers, this journal provides supporting information supplied by the authors. Such materials are peer reviewed and may be re‐organized for online delivery, but are not copy‐edited or typeset. Technical support issues arising from supporting information (other than missing files) should be addressed to the authors.

Supplementary
