# Supplementary material for: High‐Performance Li‐O2 Batteries with Trilayered Pd/MnOx/Pd Nanomembranes
Source: Adv Sci (Weinh). 2015 May 26;2(9):1500113. doi: 10.1002/advs.201500113 (PMC5115390; doi:10.1002/advs.201500113)
Supplement: Supplementary file 1 — Supplementary [file ADVS-2-0b-s001.pdf]

Copyright WILEY-VCH Verlag GmbH & Co. KGaA, 69469 Weinheim, Germany, 2015.

ADVANCED SCIENCE
Open Access

Supporting Information

for *Adv. Sci.*, DOI: 10.1002/advs.201500013

High-Performance Li-O_2 Batteries with Trilayered Pd/MnO_x /Pd Nanomembranes

Xueyi Lu, Junwen Deng, Wenping Si, Xiaolei Sun, Xianghong Liu, Bo Liu, Lifeng Liu, Steffen Oswald, Stefan Baunack, Hans Joachim Grafe, Chenglin Yan,* and Oliver G. Schmidt*

Copyright WILEY-VCH Verlag GmbH & Co. KGaA, 69469 Weinheim, Germany, 2013.

Supporting Information

High–performance Li–O_2 batteries with trilayered Pd/MnO_x/Pd nanomembranes

Xueyi Lu, Junwen Deng, Wenping Si, Xiaolei Sun, Xianghong Liu, Bo Liu, Lifeng Liu, Steffen Oswald, Stefan Baunack, Hans Joachim Grafe, Chenglin Yan,* and Oliver G. Schmidt*

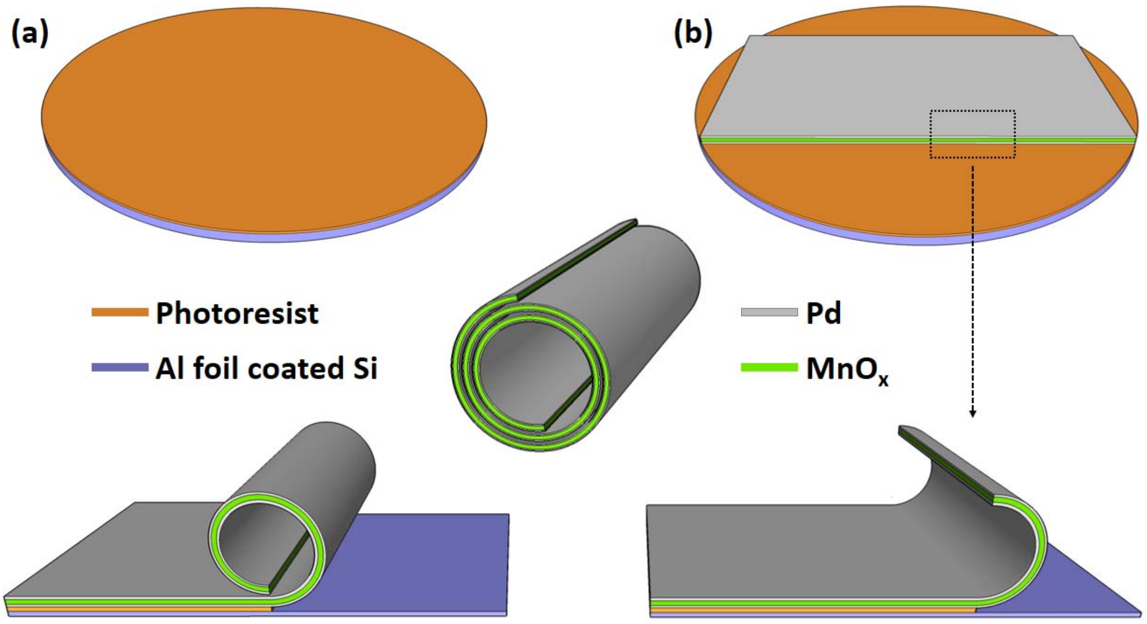

Figure S1. Schematic illustration of the preparation process of the Pd/MnO_x/Pd nanomembrane.

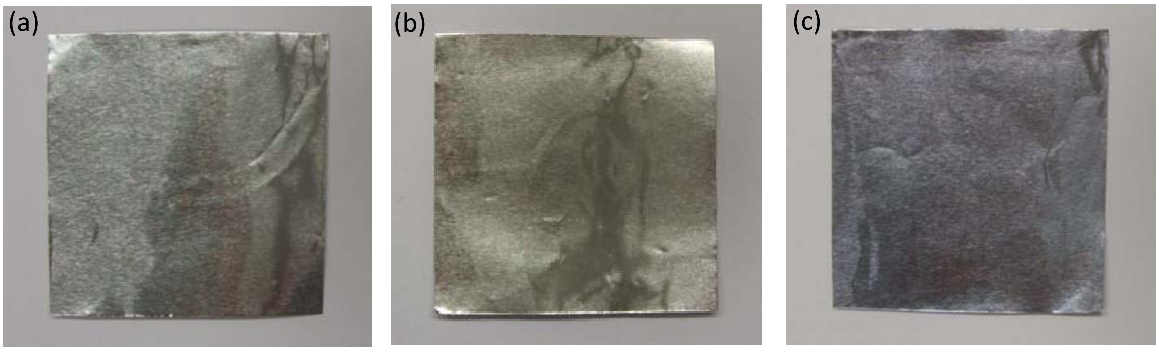

Figure S2. (a) Images of pristine Al foil, (b) Al foil after Pd deposition and (c) Al foil coated with Pd after MnO_x deposition.

We measured the percentages of Pd and MnO_x in the Pd/MnO_x/Pd nanomembranes as follows: to begin with, a cubic piece of Al foil was cut and weighted to be 130.27 mg. Then 50 nm Pd film was deposited onto the foil and it was weighted again, 131.33 mg. After that, 100 nm MnO_x was deposited onto the Pd. The final weight was measured to be 131.78 mg. Based on above data, we can get that the weights of 50 nm Pd and 100 nm MnO_x are 1.06 mg and 0.45 mg, respectively. Then the densities based on the thickness of Pd and MnO_x can be calculated to be 0.0212 mg nm^{-1}(Pd) and 0.0045 mg nm^{-1}(MnO_x), respectively. According to the value, we can obtain the percentages of Pd and MnO_x in the trilayered Pd/MnO_x/Pd nanomembrane with 6 nm Pd and 30 nm MnO_x, which are 48.51% and 51.49%, respectively. The total loading of each electrode disc is 0.3~0.5 mg cm^{-2} and the electrode consists of carbon black, catalyst and PVDF with the mass ratio of 60:20:20. We finally obtain that the net loading of Pd is 0.0291~0.0485 mg cm^{-2} which is very low.

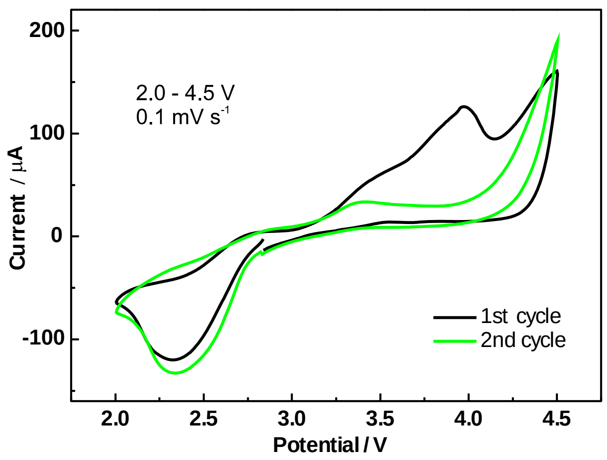

Figure S3. Cyclic voltammetry of Li–O_2 battery with Pd/MnO_x/Pd electrode at a scan rate of 0.1 mV s^{-1}.

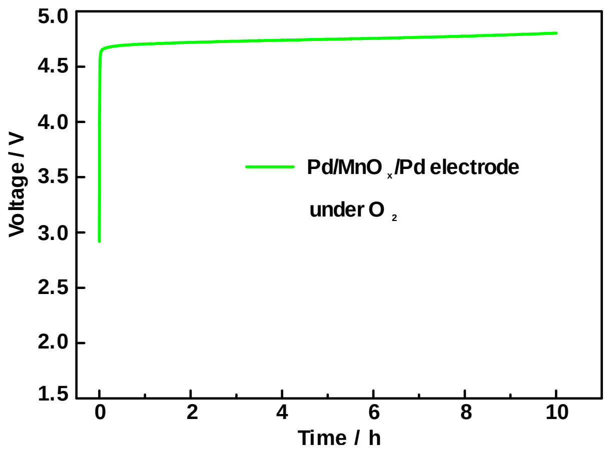

Figure S4. Galvanostatic charging profile of Li–O_2 battery with Pd/MnO_x/Pd electrode under O_2 from open circuit voltage.

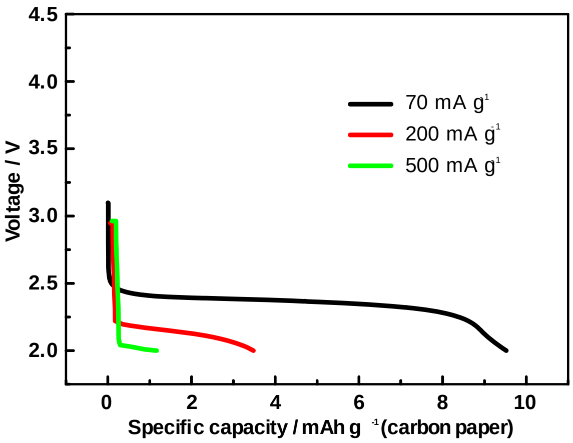

Figure S5. Discharge profile of Li–O_2 batteries with pristine carbon paper. The current density is the same as that applied to Li–O_2 batteries with Super P electrode. This profile shows that the capacity of the pristine carbon paper is quite low (below 10 mAh g^{-1}). Therefore, it is safe to neglect the contribution of the carbon paper.

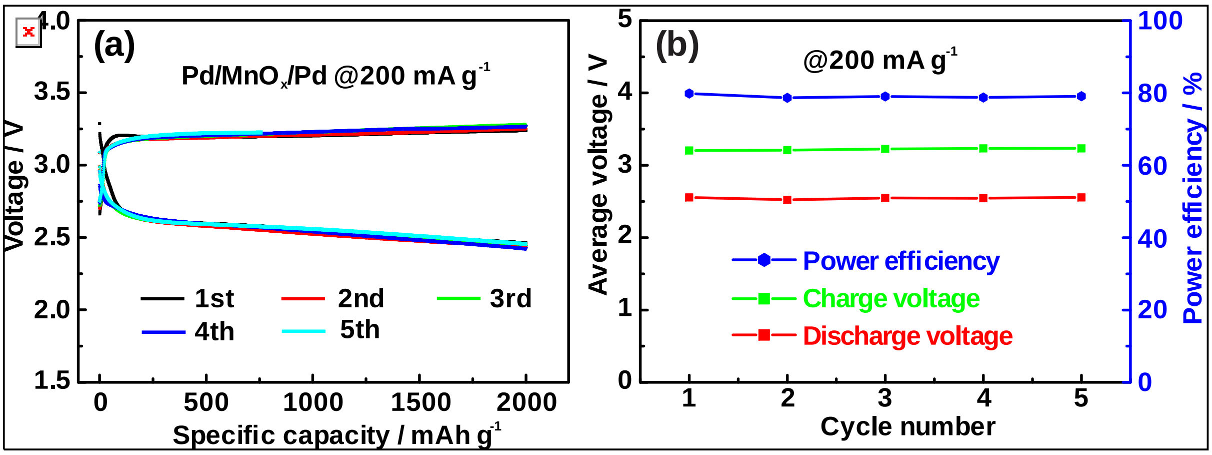

Figure S6. Discharge–charge curves and power efficiency of Li–O_2 battery with Pd/MnO_x/Pd cathode at 200 mA g^{-1} under a specific capacity limit of 2000 mAh g^{-1}.

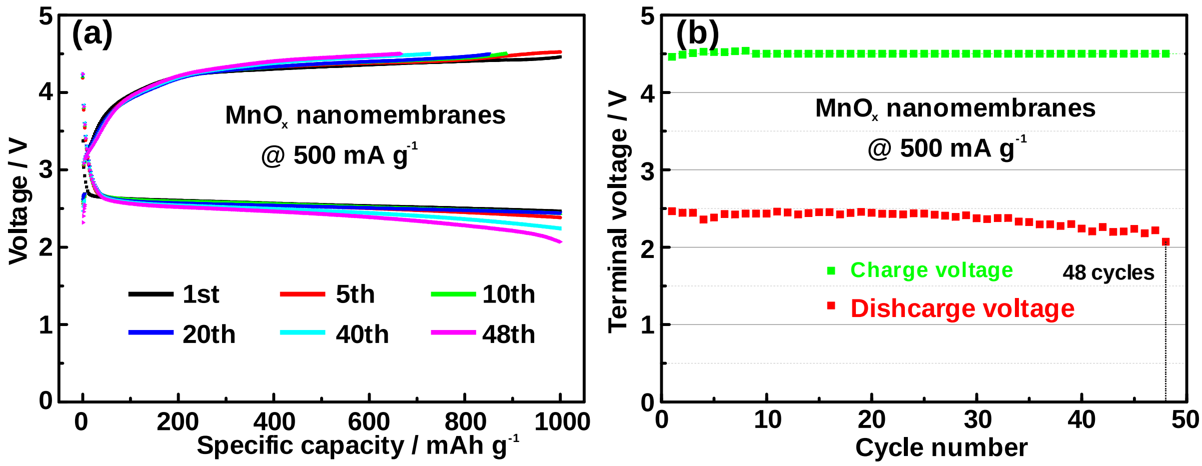

Figure S7. Discharge–charge curves and terminal voltages of Li–O_2 battery with MnO_x nanomembranes at 500 mA g^{-1} under a specific capacity limit of 1000 mAh g^{-1}.

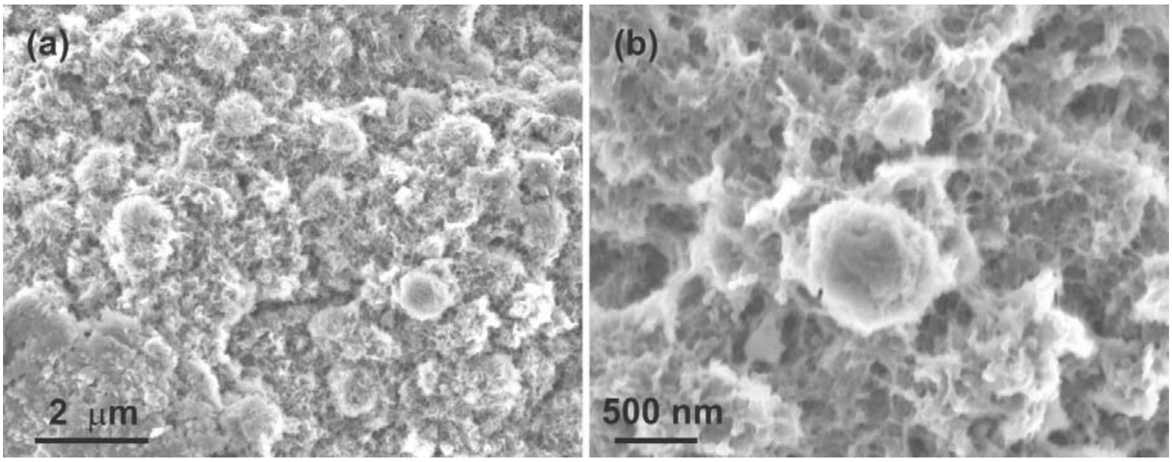

Figure S8. SEM images of the MnO_x electrode (a, b) after discharging.

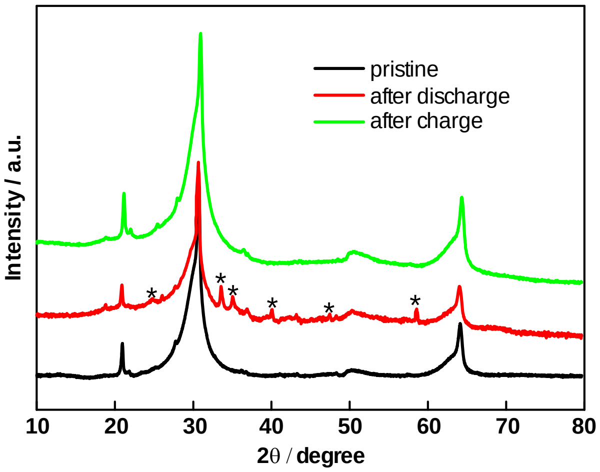

Figure S9. XRD of pristine Pd/MnO_x/Pd electrode and the electrode after the 1st discharge and charge.

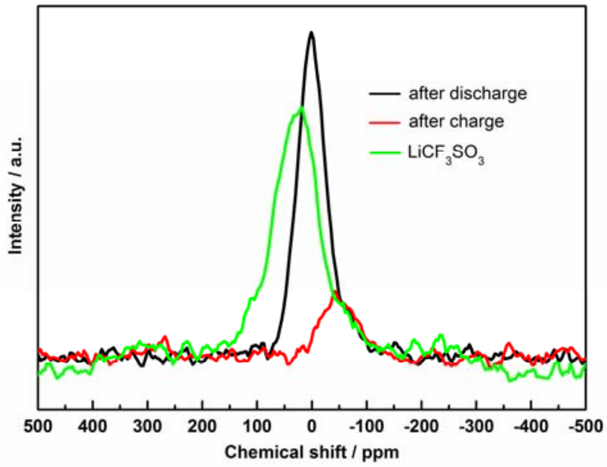

Figure S10. ^{7}Li NMR of the Pd/MnO_x/Pd electrode after discharge and charge and pure $LiCF_3SO_3$.
